# Sleep disturbances and change in multiple cognitive domains among older adults: a multicenter study of five Nordic cohorts

**DOI:** 10.1093/sleep/zsad244

**Published:** 2023-11-14

**Authors:** Marieclaire Overton, Johan Skoog, Erika J Laukka, Timothy Hadarsson Bodin, Alexander Darin Mattsson, Linnea Sjöberg, Scott M Hofer, Lena Johansson, Jenni Kulmala, Miia Kivipelto, Alina Solomon, Ingmar Skoog, Ingemar Kåreholt, Shireen Sindi

**Affiliations:** Division of Geriatric Medicine, Department of Clinical Sciences in Malmö, Lund University, Skåne University Hospital, Malmö, Sweden; Neuropsychiatric Epidemiology Unit, Department of Psychiatry and Neurochemistry, Institute of Neuroscience and Physiology, Sahlgrenska Academy at the University of Gothenburg, Gothenburg, Sweden; Aging Research Center (ARC), Department of Neurobiology, Care Sciences and Society, Karolinska Institutet and Stockholm University, Stockholm, Sweden; Stockholm Gerontology Research Center, Stockholm, Sweden; Neuropsychiatric Epidemiology Unit, Department of Psychiatry and Neurochemistry, Institute of Neuroscience and Physiology, Sahlgrenska Academy at the University of Gothenburg, Gothenburg, Sweden; Aging Research Center (ARC), Department of Neurobiology, Care Sciences and Society, Karolinska Institutet and Stockholm University, Stockholm, Sweden; Aging Research Center (ARC), Department of Neurobiology, Care Sciences and Society, Karolinska Institutet and Stockholm University, Stockholm, Sweden; Aging Research Center (ARC), Department of Neurobiology, Care Sciences and Society, Karolinska Institutet and Stockholm University, Stockholm, Sweden; Department of Neurology, Oregon Health & Science University, Portland, OR, USA; Neuropsychiatric Epidemiology Unit, Department of Psychiatry and Neurochemistry, Institute of Neuroscience and Physiology, Sahlgrenska Academy at the University of Gothenburg, Gothenburg, Sweden; Division of Clinical Geriatrics, Center for Alzheimer Research, Karolinska Institutet and Karolinska University Hospital, Stockholm, Sweden; Faculty of Social Sciences, Tampere University, Tampere, Finland; Division of Clinical Geriatrics, Center for Alzheimer Research, Karolinska Institutet and Karolinska University Hospital, Stockholm, Sweden; Ageing Epidemiology (AGE) Research Unit, School of Public Health, Imperial College London, London, UK; Theme Inflammation and Aging. Karolinska University Hospital, Stockholm, Sweden; Institute of Public Health and Clinical Nutrition, University of Eastern Finland, Kuopio, Finland; Division of Clinical Geriatrics, Center for Alzheimer Research, Karolinska Institutet and Karolinska University Hospital, Stockholm, Sweden; Ageing Epidemiology (AGE) Research Unit, School of Public Health, Imperial College London, London, UK; Theme Inflammation and Aging. Karolinska University Hospital, Stockholm, Sweden; Institute of Clinical Medicine, Neurology, University of Eastern Finland, Kuopio, Finland; Neuropsychiatric Epidemiology Unit, Department of Psychiatry and Neurochemistry, Institute of Neuroscience and Physiology, Sahlgrenska Academy at the University of Gothenburg, Gothenburg, Sweden; Aging Research Center (ARC), Department of Neurobiology, Care Sciences and Society, Karolinska Institutet and Stockholm University, Stockholm, Sweden; Division of Clinical Geriatrics, Center for Alzheimer Research, Karolinska Institutet and Karolinska University Hospital, Stockholm, Sweden; Institute of Gerontology, School of Health and Welfare, Aging Research Network – Jönköping (ARN-J), Jönköping University, Jönköping, Sweden; Division of Clinical Geriatrics, Center for Alzheimer Research, Karolinska Institutet and Karolinska University Hospital, Stockholm, Sweden; Ageing Epidemiology (AGE) Research Unit, School of Public Health, Imperial College London, London, UK

**Keywords:** sleep disturbances, cognitive domains, cognition, napping, cognitive decline, old age

## Abstract

**Study Objectives:**

We examined and compared cross-sectional and longitudinal associations between self-reported sleep disturbances and various cognitive domains in five separate Nordic European longitudinal aging studies (baseline *N* = 5631, mean age = 77.7, mean follow-up = 4.16 years).

**Methods:**

Comparable sleep parameters across studies included reduced sleep duration/quality, insomnia symptoms (sleep latency, waking up at night, and early awakenings), short and long sleep duration, and daytime napping. The cognitive domains were episodic memory, verbal fluency, perceptual speed, executive functioning, and global cognition (aggregated measure). A series of mixed linear models were run separately in each study and then compared to assess the level and rate of change in cognitive functioning across each sleep disturbance parameter. Models were adjusted for age, sex, education, hypnotic usage, depressive symptoms, lifestyle factors, cardiovascular, and metabolic conditions. By using a coordinated analytic approach, comparable construct-level measurements were generated, and results from identical statistical models were qualitatively compared across studies.

**Results:**

While the pattern of statistically significant results varied across studies, subjective sleep disturbances were consistently associated with worse cognition and steeper cognitive decline. Insomnia symptoms were associated with poorer episodic memory and participants sleeping less or more than 7–8 hours had a steeper decline in perceptual speed. In addition, daytime napping (>2 hours) was cross-sectionally and longitudinally associated with all examined cognitive domains. Most observed associations were study-specific (except for daytime napping), and a majority of association estimates remained significant after adjusting for covariates.

**Conclusion:**

This rigorous multicenter investigation further supports the importance of sleep disturbance, including insomnia, long and short sleep duration, and daytime napping on baseline cognitive functioning and rate of change among older adults. These sleep factors may be targeted in future lifestyle interventions to reduce cognitive decline.

## Graphical Abstract

**Graphical Abstract:**
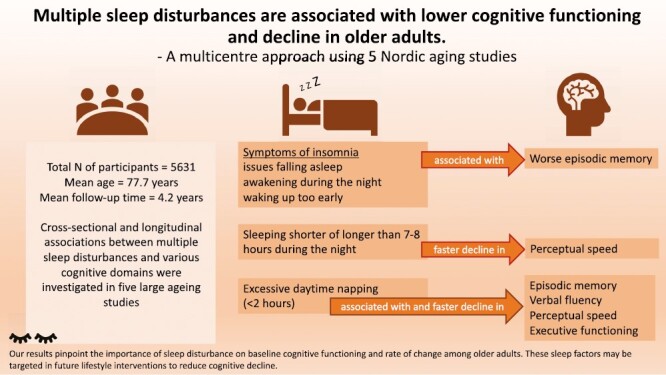

**Statement of Significance**

Data from five large Nordic European longitudinal aging studies showed that participants reporting sleep disturbances performed worse on tests of episodic memory, verbal fluency, perceptual speed, executive functioning, and global cognition compared to those not reporting sleep disturbances. The findings highlight the importance of sleep disturbances among older adults, including insomnia (sleep latency, waking up at night, and early awakenings), short and long sleep duration, and daytime napping, which may be targeted in future lifestyle interventions to improve cognition.

## Introduction

Cognitive impairment and sleep disorders are common determinants of health among older adults, and these conditions often coincide [1, 2]. While change in sleeping patterns can be a normal feature of aging, sleep disturbances in old age, e.g. too short or long sleep duration, insomnia disorder, poor sleep quality, excessive daytime sleepiness, and sleep-disordered breathing, are associated with increased risk for cognitive impairment, all-cause dementia, and Alzheimer’s disease (AD) [3–9]. This evidence suggests that the prevention of cognitive impairment, could be facilitated by identifying and targeting specific sleep parameters that increase the risk for cognitive deficits. Previous studies, however, report mixed and contradictory results regarding the nature of the cognitive impairment, e.g. memory or executive functioning, in relation to the type of sleep disturbances [10]. In addition, the few intervention studies investigating whether insomnia treatment improves cognition show varying results on a range of cognitive abilities [11–14].

Experienced prolonged sleep latency (difficulty in initiating sleep), problems with sleep maintenance (waking up at night), or terminal insomnia (early awakenings) are all signs or symptoms of insomnia [15]. They are also part of the insomnia disorder diagnosis, where at least one of the sleep symptoms must be as frequent as three times a week and a disturbance for more than three months. Moreover, they must coexist with daytime impairment in functioning and occur despite opportunities for sleep [16]. Chronic insomnia symptoms are highly prevalent among older adults [17]. Debate on what constitutes as insomnia and insomnia disorder is ongoing, and this current study will separately investigate sleep latency, waking up at night, and early awakenings as symptoms of insomnia. Cross-sectional studies with older adults show that both objective and subjective measures of insomnia are associated with worse performance in attention, long-term memory, psychomotor speed, and executive functioning [18–22]. However, several studies measuring different types of insomnia symptoms fail to find comparable associations with cognitive domains or for measures of global cognition [23–27]. Longitudinally, insomnia symptoms have been associated with cognitive decline and dementia in different adult samples globally [5, 28–30]. For instance, worse scores on comprehensive measures of cognition have been shown for subjective measures of sleep latency, sleep maintenance, and waking up too early [6, 31]. A recent study examined insomnia symptoms and failed to find longitudinal associations with worsening of memory, executive functioning and psychomotor speed, which were measured with neuropsychological testing [32]. Still, few studies have examined longitudinal change in individual cognitive domains, and often, these studies have used aggregated measurements of insomnia. Such methodological limitations compromise the interpretation of whether particular insomnia parameters increase the risk for cognitive decline more than others. Moreover, insomnia can act as a risk factor for or be a consequence of medical or psychiatric disorders. Because these factors are also related to cognition, it is desirable to control for them to avoid confounders potentially influencing the association between sleep and cognitive domains.

Approximately one-third of older adults report sleeping more, or less, than 7–9 hours of sleep per night [33]. The need for sleep may decrease somewhat with age; the National Sleep Foundation recommends older adults to sleep between 7 and 8 hours in contrast to 7–9 hours per night for younger adults [34]. The association between total sleep time and cognition is commonly described as an inverted U-shaped curve [17]; those with subjectively short or long sleep performed worse on global cognition [24, 35, 36], episodic memory, executive functioning, verbal fluency [36, 37], attention, working memory, processing speed [38], and verbal short-term memory [18]. Furthermore, studies show faster decline on global measures of cognition for short or long sleep, with follow-up times ranging from 1–10 years [24, 26, 36, 39]. Noticeably, there are considerable inconsistencies in results across studies, which may be related to differences in length of follow-up, sleep duration, and demographical factors. There is less evidence regarding change over time for specific cognitive abilities and sleep duration [36].

Daytime napping is common among older adults [40], and research on its relation to cognition has increased in recent years with varying conclusions. Napping is proposed to have both adverse and beneficial effects on cognitive functioning and decline, as well as dementia progression [21, 26, 41–43]. Contradictions in the results’ inferences are partly due to variations in napping measures and aspects such as napping duration, timing, frequency, health status, night time sleeping habits, and intentionality. For example, shorter naps are associated with improved cognitive test scores, decreased risk of cognitive decline, and dementia in comparison to longer daytime napping [43–45]. Regarding deliberation of daytime naps, one study found that unintentional nappers had poorer episodic memory than intentional nappers [46]. To date, existing research on napping in relation to specific cognitive abilities in older adults is limited.

Given the inconsistencies in the literature on how insomnia symptoms, sleep duration, and daytime napping relate to cognitive functioning, in particular to cognitive decline within specific cognitive domains, additional comprehensive research is required. Furthermore, many studies fail to routinely correct for appropriate covariates, such as depression or cardiovascular health, that could potentially explain sleep–cognition associations.

Therefore, coordinated research efforts from longitudinal aging studies, using similar measures of the most prevalent sleeping conditions (i.e. insomnia, short and long night time sleep duration, and daytime napping), cognitive domains, and relevant covariates, are required to rigorously compare and synthesize cross-sectional and longitudinal associations between sleep and cognition in later life. Consequently, the aim of this study was to examine cross-sectional and longitudinal associations between several self-reported sleep disturbances and cognitive domains while applying a coordinated analytic approach based on identical statistical models using data from five longitudinal aging studies.

## Methods

### Analytical approach

The current study used a coordinated analysis approach, which as described by Hofer et al., harmonizes the measurements (sleep and cognition) across studies and facilitates the comparisons of the associations across studies [47]. This is achieved by analyzing data from each study separately using identical statistical models and evaluating the pattern of results to examine similarities in research findings and potential sources of heterogeneity. Coordinated analysis refers to the simultaneous analysis of multiple datasets, with the goal of improving the consistency and reliability of the results when exact measurements across studies are not available and also when there are differences in study designs that limit data pooling for a combined statistical analysis. By conducting coordinated analysis, researchers can improve the external validity and generalizability of findings across different populations or contexts; increase statistical power by pooling data from multiple sources; enhance the ability to detect and correct for biases or errors in individual datasets; and facilitate comparisons and contrasts between datasets, potentially leading to new insights. Results from a coordinated analysis can enhance transparency and reproducibility of research by promoting data sharing and collaboration in ways that yield new results from many studies. A coordinated analysis differs from an integrative data analysis (pooling raw data from multiple studies to create a single, larger dataset for analysis), which requires exact harmonization (usually identical measures) and combines the datasets of different studies into a merged dataset prior analysis, and produces one rather than several sets of results [48]. The methodological procedures of coordinated analyses have been described earlier and used effectively to facilitate the replication of findings and increase confidence in results in other research areas [49, 50].

### Study descriptions and participants

This study used data from five Nordic European longitudinal population-based studies on aging, four from Sweden, The Gothenburg H70 Birth Cohort Studies (H70), two studies from the Swedish National Study on Aging and Care (SNAC) and the Kungsholmen Project (KP), and one from Finland, The Cardiovascular Risk Factors, Aging and Dementia (CAIDE). Participants provided written consent, and all studies were approved by their local ethics committee. Participants with dementia or Mini Mental State Examination (MMSE) under 24 at baseline were excluded from all studies.

#### The Gothenburg H70 Birth Cohort Studies (H70).

The H70 studies are representative birth cohorts of older populations in Gothenburg, Sweden. All samples were systematically selected from the Swedish Population Register based on birth dates in order to yield representative samples. In this study, we included a birth cohort born 1930, which was examined in 2000, 2005, 2009, and 2015. Baseline was defined as the first-time participants had data on both cognitive tests and sleep.

In 2000–2002, 70-year-olds living in Gothenburg and born in 1930 on days 3, 6, 12, 18, 21, 24, or 30 every month were examined (*N* = 524; eligible response rate: 69.6%). These days were chosen to ensure a representative sample of certain number of participants. Notably, only half of the participants underwent cognitive testing during this examination (*n* = 215). After excluding those with dementia (*n* = 3) or MMSE < 24 (*n* = 5), 207 were included as baseline sample.

In 2005–2007, a follow-up was conducted at age 75. In addition, the sample was extended to also include persons born on days 2, 5, 11, 16, 20, or 27. In total, 767 accepted to participate (eligible response rate: 64.1%). Among these, 521 had both sleep and cognitive data for the first time. After excluding those with dementia (*n* = 17) or MMSE < 24 (*n* = 17), 487 were included as baseline sample.

In 2009–2011, a follow-up was conducted at age 79 (*N* = 580; eligible response rate: 61.1%). Among these, 88 had both sleep and cognitive data for the first time. After excluding those with dementia (*n* = 6) or MMSE < 24 (*n* = 1), 81 were included as baseline sample.

In 2015–2017, a follow-up was conducted at age 85 (*N* = 416; eligible response rate: 61.9%). Among these, 43 had both sleep and cognitive data for the first time. After excluding those with dementia (*n* = 33) or MMSE < 24 (*n* = 3), 7 were included as baseline sample.

The baseline used in this study thus included 782 individuals (207 from 2000, 487 from 2005, 81 from 2009, and 7 from 2015).

#### Swedish National Study on Aging and Care (SNAC).

SNAC is a Swedish national aging study that started in 2001 and includes four participating geographical areas: SNAC-Blekinge, SNAC Nordanstig, SNAC Kungsholmen (SNAC-K), and SNAC-Good aging in Skåne (SNAC-GÅS). SNAC-K and SNAC-GÅS contributed with data to this study due to their matching protocols, specifically with regard to the cognitive testing, which is identical both at baseline and all follow-ups between these sites. Participants aged 60, 66, 72, 78, 81, 84, 87, 90, 93, 96, and 99 years were randomly selected through the Swedish population registry to take part in the studies at each respective study site. Reexamination occurred every 6 years until age 78, after which reexamination occurred every 3 years. In both the current SNAC-studies, participants aged 66 years and younger were excluded to closer match participant age in the other studies.

In SNAC-GÅS, individuals living in rural and urban parts of southern Sweden (Malmö and three smaller towns in Skåne) were invited. A total of 60% (*n* = 2391) agreed to participate and 1548 of those were 72 years and older born between 1908 and 1931. After exclusion of those with dementia (*n* = 100) or MMSE < 24 (*n* = 218), 1133 participants were included in the study sample, with up to four reexaminations: I (*n* = 544), II (*n* = 632), III (*n* = 361), IV (*n* = 269), and V (*n* = 147).

People living in the area of Kungsholmen in central Stockholm were invited to take part in SNAC-K. In total 3363 (73%) individuals participated, of which 2059 were 72 years or older born between 1898 and 1931. After excluding those with dementia (*n* = 306), developmental disorder (*n* = 3), MMSE < 24 (*n* = 47), or missing data on cognitive status (*n* = 10), we further excluded those with missing data on sleep (*n* = 18) or the neuropsychological battery (*n* = 185), leaving 1490 participants to be included in the study sample. Participants in the present sample were reexamined up to five times: follow-up I (*n* = 671), II (*n* = 765), III (*n* = 533), and IV (*n* = 232).

#### The Kungsholmen Project (KP).

All inhabitants of the area of Kungsholmen in central Stockholm who were ≥ 75 years on October 1, 1987 (*n* = 2368), were invited to participate in the KP study. In this initial screening phase, 1810 (76%) individuals participated. In wave 2, where the neuropsychological battery was introduced, 668 individuals participated. After excluding those with dementia (*n* = 225) or MMSE < 24 (*n* = 98), we further excluded those with missing data on sleep (*n* = 2) or the neuropsychological battery (*n* = 20), leaving 323 participants to be included in the study sample. Participants were followed up every 3 years and reexamined at follow-up I (*n* = 192), II (*n* = 121), and III (*n* = 80).

#### The Cardiovascular Risk Factors, Aging and Dementia study (CAIDE).

The CAIDE study was conducted in Finland. Midlife assessments took place within the North Karelia Project and FINMONICA study, during one of the following years: 1972, 1977, 1982, or 1987. At baseline, the participation rates ranged between 82% and 90%. A random sample of 2000 survivors (in 1998), living in the cities of Kuopio and Joensuu (age 65–79 years), were invited to the first reexamination, that is used as baseline in this study. A total of 1449 individuals participated, of which 1409 completed the neuropsychological tests. Participants were invited for a second reexamination between 2005 and 2008, during which 1426 individuals were alive and living in the same region. Of these, 909 individuals accepted to participate, and 852 completed the neuropsychological tests. In total, 1511 individuals participated in at least one reexamination, and 750 participated in both. Mean ages were 71.0 years (SD = 3.9) at the first reexamination and 78.6 years (SD = 3.7) at the second reexamination. For consistency with the other cohorts included in this study, the first reexamination (when mean age was 71 years was used as the baseline, while the second reexamination was used as the follow-up). Participants with missing data on sleep or other covariates were excluded, as were individuals with dementia at the first reexamination (late life), leaving 1115 participants for analyses.

## Measurements

### Sleep variables

Measurements of sleep were self-reported either by interview or by questionnaire filled out by the participant. Sleep questions from each study were chosen based on their similarity, availability, and opportunity for close comparisons at the narrow construct level. When sleep questions were dichotomized (yes/no) in one study but were scale in another, the scale was made binary to appropriately match the yes/no question. The following sleep parameters were considered: reduced sleep duration/quality, insomnia symptoms (sleep latency, waking up at night, early awakenings), extensive daytime napping, and sleep duration (short and long). A measurement of general subjective disturbed sleep, reduced sleep duration/quality, was based on a combined question from the Comprehensive Psychiatric Rating Scale [51]. The participant’s experience of reduction in the duration and/or in the depth of sleep in comparison with how they normally sleep was registered and was available in H70, both SNAC-studies and in the Kungsholmen study. Formulation of the three separate insomnia questions differed slightly between studies (see Table 1 for precise formulation) and were available in the H70 study, both SNAC-studies and CAIDE (early awakenings only).

**Table 1.** Descriptions of sleep parameters in the studies H70, SNAC-GÅS, SNAC-K, KP, and CAIDE

Sleep variable	Study	Item description
Reduced sleep duration/quality sleep	H70, SNAC-GÅS, SNAC-K, and KP	Item from the Comprehensive Psychopathological Rating Scale (CPRS): The item measured “a subjective feeling of reduced duration or depth of sleep compared with the subject’s own normal pattern”: (0–1) I sleep as normal (2–3) moderate difficulties in initiating sleep, or shorter, lighter, or disturbed sleep (4–5) reduced sleep with at least 2 hours per night or early awakenings without external influence (6) less than 2–3 hours sleep per night The question was dichotomized as response alternatives 0–1 versus 2–6
Sleep latency	H70, SNAC-GÅS, and SNAC-K	Do you have trouble falling asleep? 1) Yes 2) No
Waking up at night	SNAC-K	Do you wake up during the night? 1) Yes 2) No
	GÅS	Do you wake up multiple times during the night? 1) Yes 2) No
	H70	Do you wake up one or multiple times per night? 1) Yes 2) No
Early awakenings	H70	Do you wake up early in the morning? 1) Yes 2) No
	SNAC-GÅS and SNAC-K	Do you wake up early? 1) Yes 2) No
	CAIDE	Item on sleep in the Beck Depression Inventory (BDI): 1) I sleep as well as before 2) When I wake up in the morning, I’m more tired than before 3) I wake up 1–2 hours earlier than usual and it is hard for me to fall back asleep 4) I wake up early every morning and I am not able to get more than 5 hours of uninterrupted sleep The question was dichotomized as response alternatives 1–2 indicating non early awakenings and 3–4 indicating early awakenings
Sleep duration	SNAC-GÅS and H70	On average, how many hours do you sleep per night? Response was in hours and categorized as following: short sleep <6 hours, normal sleep = 7–8 hours, and long sleep >9 hours
Daytime napping	SNAC-GÅS and SNAC-K	Do you feel tired and sleep more than 2 hours during the day? 1) Yes 2) No
	H70	Do you rest or sleep during the day? 1) No 2) Do not sleep, rests 0–1 hours 3) Do not sleep, rests more than 1 hour 4) Sleeps 0–1 hours per day 5) Sleeps 1–2 hours per day 6) Sleeps more than 2 hours per day The question was dichotomized as response alternatives 0–5 versus 6

All sleep questions are self-reported by questionnaire or by interview.

Abbreviations: SNAC-K: Swedish National Study on Aging and Care-Kungsholmen; SNAC-GÅS: Swedish National Study on Aging and Care – Good Aging in Skåne; H70: The Gothenburg H70 Birth Cohort Studies; CAIDE: The Cardiovascular Risk Factors, Aging and Dementia study; KP: The Kungsholmen Project.

Sleep latency was measured through participant’s experience of issues of falling asleep. Waking up at night was measured through participant’s experience of waking up once or multiple times per night (frequency of awakenings was study specific). In H70 and SNAC, early awakenings were assessed by asking whether the participant woke up early, and in CAIDE, a combined question from the Beck Depression Inventory [52] was used. Extensive daytime napping, which is more strongly associated with cognitive decline in contrast to short daytime napping [43–45], was assessed by asking the participant if they slept for 2 or more hours during the day and was available in the H70 study and in SNAC-studies. Sleep duration was available in H70 and in SNAC-GÅS where participants on average were asked how long they slept per night, and response was categorized: short sleep ≤ 6 hours, normal sleep = 7–8 hours and long sleep ≥ 9 hours based on the recommendations for older adults night time sleep [34]. See Table 1 for formulation of the sleep questions, and how they were categorized in each study.

### Cognitive variables

Description of the cognitive testing procedures and the specific tests used in each study are found elsewhere [53–57]. Cognitive tests were available in each of the following cognitive domains: episodic memory (free recall and recognition of words, objects, or pictures), perceptual speed (figure identification, digit cancelation, or pattern comparison), verbal fluency (animals, professions, or food items), and executive functioning (SRB2 logical reasoning, Trail Making Test B, or WAIS III Block Design). All scores were standardized based on the baseline mean and standard deviation of their respective sample. When more than one test was available for a domain, a composite score was computed by taking the mean of the included standardized scores. Finally, a composite score of all four domains was created, taking the mean of the domain-specific scores, to represent global cognition.

### Covariates and dementia diagnosis

Procedures were standardized, and assessments remained the same between examination waves. In all studies, participants filled out questionnaires or were interviewed regarding their medical history, sociodemographic factors, health status and behavior, and psychiatric symptoms. Detailed descriptions of the dementia diagnosis for each study have been described in detail [29, 53, 54, 58, 59]. In all five studies, dementia diagnoses were determined using the Diagnostic and Statistical Manual of Mental Disorders (DSM) criteria (DSM-IV in GÅS, SNAC-K and CAIDE; DSM-III in H70 and KP). Hypnotic use was based on drug registers and blood pressure was measured with a sphygmomanometer. Continuous variables: age, education (years in formal school), depressive symptoms (based on two questions regarding low mood on the Comprehensive Psychopathological Rating Scale [60] and Beck depression Inventory [52]), alcohol consumption (grams of ethanol per week or categorically with no/occasional consumption, light/moderate or heavy drinking), systolic blood pressure (in mm Hg), and number of cardiovascular and metabolic conditions (ischemic or hemorrhagic stroke, myocardial infarction, atrial fibrillation, heart failure, or diabetes). Categorical variables: sex (female vs. male), use of hypnotics (yes vs. no), cohabitant status (living alone vs. cohabiting), smoking (current smoker vs. former and never smoked), physical activity (sedentary vs. light to moderate), alcohol consumption (3 categories: zero or occasional consumption, light to moderate. or heavy drinking). Venous blood samples were attained, and DNA was extracted to determine *APOE* genotype (ε4 vs. no ε4). See Table 2 for overview of baseline covariates and characteristics of participants in each study.

**Table 2.** Baseline characteristics of participants across H70, SNAC-GÅS, SNAC-K, KP, and CAIDE studies

Variables	H70 (*n* = 782)	SNAC-GÅS (*n* = 1133)	SNAC-K (*n* = 1490)	KP (*n* = 323)	CAIDE (*n* = 1115)
Number of assessments, M (SD)	1.86 (0.93)	2.52 (1.52)	2.54 (1.36)	2.22 (1.16)	1.58 (0.49)
Mean follow-up time in years, M (SD)	4.38 (4.84)	4.66 (4.60)	5.28 (4.53)	3.88 (3.70)	4.83 (4.16)
Demographics					
Age, M (SD)	74.2 (2.92)	80.7 (6.24)	80.3 (6.68)	84.3 (5.27)	71.0 (3.88)
Sex, female, *n* (%)	462 (59.1)	678 (59.8)	989 (66.4)	263 (81.4)	681 (61.5)
Education years, M (SD)	10.4 (4.10)	9.28 (3.42)	11.0 (4.04)	8.85 (2.66)	8.99 (3.48)
Registered use of hypnotics					
Registered users *n* (%)	85 (12.4)	221 (19.5)	350 (23.5)	91 (28.2)	105 (9.42)
Depressive symptoms					
CPRS/BDI, M (SD)	0.94 (1.46)	0.57 (1.20)	0.30 (0.80)	0.46 (0.94)	0.51 (0.92)
Life-style factors					
Living alone, *n* (%)	280 (35.9)	613 (54.5)	912 (61.4)	243 (75.2)	426 (38.2)
Current smoker, *n* (%)	93 (11.9)	104 (9.17)	162 (10.9)	104 (32.2)	410 (36.8)
Not physically active, *n* (%)	154 (21.5)	187 (16.5)	487 (32.7)	230 (71.2)	138 (12.4)
Alcohol continuous measure, M (SD)					
Alcohol in grams per week	54.2 (74.8)	21.6 (46.3)	—	—	—
Units per week	—	—	—	—	2.15 (4.16)
Alcohol categorical measure, *n* (%)					
No alcohol or occasional consumption	—	—	607 (40.9)	—	—
Light to moderate	—	—	676 (45.6)	—	—
Heavy drinking	—	—	200 (13.5)	—	—
Metabolic and cardiovascular conditions					
CVDs (0-5), M (SD)	0.41 (0.72)	0.57 (0.85)	0.88 (1.06)	—	0.39 (0.75)
CVDs (0-3), M (SD)	—	—	—	0.32 (0.52)	—
Systolic blood pressure, M (SD)	159.5 (21.2)	153.4 (24.4)	146.9 (20.1)	156.6 (22.2)	145.4 (23.0)
*APOE* any ε4, *n* (%)		—			
Yes	200 (26.1)	—	388 (28.0)	59 (29.2)	379 (34.7)
No	—	—	998 (72.0)	143 (70.8)	713 (65.3)
Sleep disturbances					
Reduced sleep duration/quality (CPRS), *n* (%)	234 (30.0)	108 (9.53)	248 (16.6)	126 (39.0)	—
Sleep latency (problems falling asleep), *n* (%)	227 (29.1)	297 (26.6)	161 (10.8)	—	—
Waking up at night, *n* (%)	316 (40.9)	238 (21.0)	268 (18.0)	—	—
Early awakenings, *n* (%)					
Sleep duration, *n* (%)					
Normal sleep (7–8 hours)	676 (86.4)	666 (59.7)	—	—	—
Short sleep (<6 hours)	57 (7.33)	329 (29.5)	—	—	—
Long sleep (>9 hours)	49 (6.30)	120 (10.8)	—	—	—
Daytime napping, *n* (%)	4 (0.58)	120 (10.8)	30 (2.02)	—	—
Cognitive variables					
MMSE, M (SD)	27.9 (1.35)	26.9 (1.68)	28.5 (1.50)	27.1 (1.55)	26.6 (1.42)
Global cognition, *z*-score, M (SD)	−0.06 (0.58)	−0.002 (0.66)	−0.030 (0.70)	−0.011 (0.72)	0.00 (0.89)
Episodic memory, M (SD)					
Word recall (16 words)	—	6.02 (2.11)	6.26 (2.28)	—	—
Word recall (12 words)	—	—	—	5.17 (1.62)	—
Object recall (12 objects)	7.31 (1.88)	—	—	—	4.99 (1.57)
Word recognition (16 words)	—	11.3 (3.11)	11.3 (3.11)	—	—
Word recognition (12 words)	—	—	—	8.67 (2.31)	—
Thurstone picture memory test	20.78 (4.41)	—	—	—	—
Category fluency, M (SD)					
Animals	21.8 (6.14)	18.0 (5.5)	18.8 (5.96)	—	20.5 (5.99)
Professions	—	13.2 (4.5)	13.9 (5.05)	—	—
Food items	—	—	—	17.4 (6.61)	-
Perceptual speed, M (SD)					
Figure identification	24.0 (6.17)	—	—	—	—
Digit cancelation	—	15.0 (3.9)	15.8 (3.86)	—	—
Pattern comparison	—	11.1 (3.39)	11.8 (3.50)	—	—
Stroop 1	—	—	—	—	25.3 (7.32)
Executive function, M (SD)					
Logical reasoning	15.3 (4.68)	—	—	—	—
TMT-B, seconds	—	41.5 (25.6)	37.7 (22.4)	—	—
Block design	—	—	—	13.11 (5.31)	—
Stroop 3	—	—	—	—	101.7 (71.6)

Abbreviations: *APOE*: apolipoprotein E; CVD: cardiovascular disease; CPRS: Comprehensive Psychiatric Rating Scale; BDI: Beck’s Depression Inventory; TMT: Trail Making Test; SRB2, MMSE: Mini Mental State Examination; SNAC-K: Swedish National Study on Aging and Care- Kungsholmen; SNAC-GÅS: Swedish National Study on Aging and Care – Good Aging in Skåne; H70: The Gothenburg H70 Birth Cohort Studies; CAIDE: The Cardiovascular Risk Factors, Aging and Dementia study; KP: The Kungsholmen Project.

### Statistical analyses

As studies differed in the sleep and cognitive variables, coordinated analysis was performed across studies on each variable or narrow factor combination, permitting cross-study comparison of these common variable sets at the narrow construct level. To enable qualitative comparisons between study results, test scores for each domain were converted to *z*-scores. The associations between sleep disturbances and cognitive level and change were analyzed using a series of linear mixed effects models, taking into account the dependence of repeated examinations of the participants. In each study, identical models were fit for each of the five cognitive domains; episodic memory, perceptual speed, verbal fluency, executive functioning, and global cognition in combination with each of the available sleep parameters; reduced sleep duration/quality, sleep latency, waking up at night, early awakenings, sleep duration, and daytime napping.

For cross-sectional associations, i.e. associations between baseline sleep disturbances and level of cognitive performance, baseline sleep disturbance was included as a fixed effect. A time and sleep disturbance interaction was included as a fixed effect in the models to assess potential associations between sleep and longitudinal changes in cognition. For each domain and sleep combination, a set of five models were run adjusting for the following covariates: model 1 was adjusted for demographic factors (age, sex, education,) model 2 for demographic factors and hypnotics, model 3 for demographic factors, hypnotics, and depressive symptoms, model 4 for demographic factors, hypnotics, depressive symptoms, and lifestyle factors (smoking, living alone, alcohol consumption, physical inactivity), and model 5 for demographic factors, hypnotics, depressive symptoms, lifestyle factors, and metabolic and cardiovascular conditions (stroke, myocardial infarction, atrial fibrillation, diabetes, heart failure, and hypertension).

All statistical analyses were two-sided, with *p* = 0.05 as the threshold for statistical significance. To facilitate result presentation and interpretation, we report the corresponding β-coefficient estimates from the mixed linear models in each study for the observed significant estimates from the cross-sectional (level) and longitudinal (slope) associations from the partial (basic model) and fully adjusted models. Statistical procedures were run separately in each study using either STATA version 16 and 17 (SNAC-K, Kungsholmen, and CAIDE), R (H70), or SPSS version 28 (SNAC-GÅS).

## Results

### Descriptive statistics

Baseline descriptive statistics across all studies are presented in Table 2.

Comparisons of baseline characteristics between the studies revealed that women were slightly overrepresented in all studies. The CAIDE study had the youngest participants (Mean age = 71.0) and the KP study the oldest (M age = 84.3). The SNAC-K participants had the most years of education (M = 11.0) and the highest MMSE scores (M = 28.5). Mean follow-up time ranged between 3.88 (KP) and 5.28 years (SNAC-K), and mean number of assessments was 2.14 examinations per participant across all studies. Hypnotics usage varied between 9.4% and 28.2% across studies, where KP with the oldest participants had the largest intake. Participants in the H70 study reported slightly more disturbances (sleep latency, waking up at night, and early awakenings) than in the SNAC-studies (notably, KP and CAIDE did not have instruments to measure these parameters). For reduced sleep duration/quality and early awakenings, CAIDE reported the highest frequency of sleep disturbances, with over 40% reporting waking up early.

### Mixed model results

**Figure 1.**
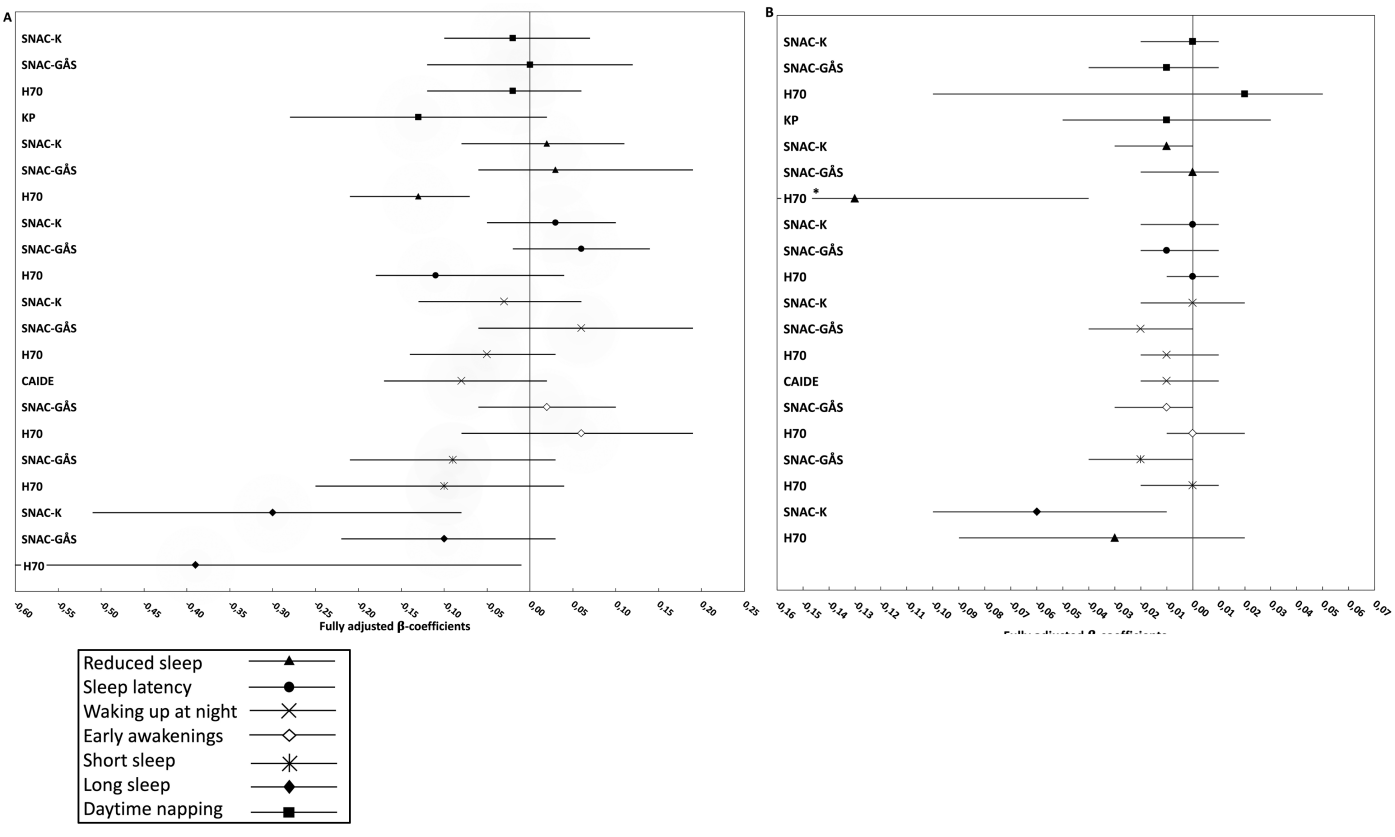
(A) Forest plot showing cross-sectional associations, with β-coefficients for global cognition across studies for each sleep parameter. (B) Forest plot showing longitudinal associations β-coefficients for global cognition across studies for each sleep parameter. Global cognition scores are composite scores (*z*-scores) of cognitive domains that include episodic memory, perceptual speed, verbal fluency, and executive functioning. Abbreviations: SNAC-K: Swedish National Study on Aging and Care – Kungsholmen; SNAC-GÅS: Swedish National Study on Aging and Care-Good Aging in Skåne; H70: The Gothenburg H70 Birth Cohort Studies; CAIDE: The Cardiovascular Risk Factors, Aging and Dementia study; KP: The Kungsholmen Project.

A consistent pattern of negative relationships between sleep disturbances and cognitive performance was observed. Of the total number of sleep–cognition associations, across studies, produced by the basic mixed linear models (model 1, controlling for demographics) and fully-adjusted (model 5, controlling for all covariates), 31.7% of the cross-sectional and 13.4% of the longitudinal associations were significant, respectively. All of the significant estimates showed that sleep disturbances were associated with lower cognitive functioning and faster cognitive decline, with no significant results in the opposite direction. For a visual overview of the results, associations for global cognition for each measured sleep disturbances, in each study, are presented in forest plots (Figure 1, A and B).

Although, the significant results were study-specific, with few studies observing corresponding sleep-cognition associations, some patterns emerged. In the models corrected for demographics, lifestyle, hypnotic usage, depressive symptoms, and metabolic and cardiovascular conditions (fully adjusted models), few (*n* = 8, 8.0%) cross-sectional and longitudinal associations for insomnia symptoms and cognitive measures were found. Participants from multiple studies reporting sleeping for longer than 9 hours per night or napping for 2 or more hours per day exhibited significantly lower scores and faster cognitive decline on tests measuring a range of cognitive abilities. In the fully adjusted models, perceptual speed estimates evoked the most significant cross-sectional and longitudinal results across studies, followed by episodic memory and executive functioning, whereas verbal fluency evoked the least. When further adjusting for *APOE*, all significant sleep–cognition associations remained, and estimates were consistent with the *APOE* unadjusted analyses (data not shown).

Unless otherwise stated, all results presented below are from fully adjusted models. Cross-sectional associations are reported first, i.e. between-subjects cognitive comparison (level), following longitudinal associations, i.e. within-subjects cognitive performance over time (slope), for each sleep disturbance individually. All significant cross-sectional and longitudinal results are presented in Tables 3 and 4, respectively.

**Table 3.** Mixed model coefficient estimates for cross-sectional associations with significant results for sleep parameter and cognitive domain in each study. Comparison of estimates from two models, one basic model and one fully adjusted model

	Cognitive domains									
	Episodic memory		Verbal fluency		Perceptual speed		Executive functioning		Global cognition	
Sleep parameter (studies with parameter)	*n*	Estimation (95% CI)	n	Estimation (95% CI)	*n*	Estimation (95% CI)	*n*	Estimation (95% CI)	*n*	Estimation (95% CI)
*Reduced sleep duration/quality (available in H70, GÅS, SNAC-K, KP)*										
KP										
Basic model	323	−0.10 (−0.28; 0.08)	307	−**0.23 (**−**0.44;** −**0.02)**		NA	314	−0.19 (−0.39; 0.02)	323	−**0.18 (**−**0.33;** −**0.04)**
Fully adjusted model	323	−0.06 (−0.26; 0.13)	307	−0.08 (−0.30; 0.13)		NA	314	−0.17 (−0.39; 0.05)	323	−0.13 (−0.28; 0.02)
*Sleep latency (available in H70, GÅS, SNAC-K)*										
H70										
Basic model	754	−**0.13 (**−**0.22;** −**0.03)**	778	−**0.11 (**−**0.21;** −**0.01)**	772	−**0.11 (**−**0.18;** −**0.03)**	746	−**0.18 (**−**0.30;** −**0.05)**	783	−**0.14 (**−**0.21;** −**0.07)**
Fully adjusted model	617	−**0.12 (**−**0.24;** −**0.01)**	629	−0.11 (−0.24; 0.01)	626	−0.08 (−0.17; 0.00)	618	−0.09 (−0.24; 0.06)	632	−**0.13 (**−**0.21;** −**0.05)**
*Waking up at night (available in H70, GÅS, SNAC-K)*										
H70										
Basic model	748	−**0.10 (**−**0.18;** −**0.01)**	767	−**0.19 (**−**0.28;** −**0.10)**	764	−**0.08 (**−**0.14;** −**0.01)**	740	−0.09 (−0.21; 0.03)	772	−**0.13 (**−**0.19;** −**0.06)**
Fully adjusted model	616	−0.05 (−0.15; 0.04)	628	−**0.19 (**−**0.30;** −**0.08)**	625	−0.06 (−0.13; 0.01)	617	−0.09 (−0.16; 0.10)	632	−**0.11 (**−**0.18;** −**0.04)**
*Early awakenings (available in H70, GÅS, SNAC-K, CAIDE)*										
H70										
Basic model	754	−**0.19 (**−**0.30;** −**0.09)**	778	−**0.14 (**−**0.25;** −**0.01)**	772	−**0.09 (**−**0.17;** −**0.01)**	746	−**0.17 (**−**0.32;** −**0.03)**	783	−**0.13 (**−**0.21;** −**0.06)**
Fully adjusted model	617	−**0.13 (**−**0.25;** −**0.01)**	629	−0.09 (−0.22; 0.04)	626	0.00 (−0.09; 0.09)	618	−0.07 (−0.23; 0.09)	632	−0.05 (−0.14; 0.03)
CAIDE										
Basic model	1750	−0.01 (−0.11; 0.10)	1750	−0.06 (−0.17; 0.04)	1708	0.08 (−0.02; 0.19)	1691	0.01 (−0.10; 0.11)^*^	1754	−**0.10 (**−**0.19;** −**0.01)**
Fully adjusted model	1750	0.02 (−0.10; 0.11)	1750	−0.04 (−0.15; 0.10)	1708	0.06 (−0.04; 0.17)	1691	−0.03 (−0.13; 0.08)	1754	−0.08 (−0.17; 0.02)
*Sleep duration (available in H70 and GÅS)*										
Short sleep										
H70										
Basic model	751	−**0.26 (**−**0.42;** −**0.09)**	775	0.01 (−0.17; 0.18)	769	0.02 (−0.15; 0.10)	743	0.02 (−0.21; 0.24)	780	−0.08 (−0.20; 0.04)
Fully adjusted model	614	-0.11 (-0.30; 0.07)	626	0.14 (-0.06; 0.35)	623	0.06 (−0.08; 0.20)	615	0.08 (−0.17; 0.33)	629	−0.06 (−0.08; 0.19)
Long sleep										
H70										
Basic model	751	−0.08 (−0.25; 0.09)	775	−0.09 (−0.27; 0.10)	769	−0.11 (−0.25, 0.02)	743	−0.03 (−0.21, 0.27)	780	−0.08 (−0.20; 0.05)
Fully adjusted model	614	−0.11 (−0.31; 0.09)	626	−0.02 (−0.24; 0.21)	623	−**0.20 (**−**0.35;** −**0.05)**	615	−0.01 (−0.28; 0.27)	629	−0.10 (−0.25; 0.04)
GÅS										
Basic model	1053	−**0.18 (**−**0.35;** −**0.02)**	1100	−**0.18 (**−**0.34;** −**0.02)**	1042	−**0.19 (**−**0.35;** −**0.03)**	783	**0.28 (0.07; 0.50)** ^*^	1037	−**0.20 (**−**0.32;** −**0.09)**
Fully adjusted model	1053	−0.01 (−0.03; 0.04)	1100	−0.06 (−0.24; 0.12)	1042	−0.10 (−0.27; 0.07)	783	0.13 (−0.09; 0.34)^*^	1037	−0.09 (−0.21; 0.03)
Daytime napping (available in H70, GÅS, SNAC-K)										
H70										
Basic model	683	−**0.74 (**−**1.31;** −**0.18)**	699	−**0.56 (**−**1.13;** −**0.01)**	696	−**0.63 (**−**1.07;** −**0.18)**	681	−0.58 (−1.48; 0.33)	704	−**0.40 (**−**0.78;** −**0.02)**
Fully adjusted model	616	−**0.70 (**−**1.28;** −**0.12)**	639	−0.52 (−1.10; 0.05)	625	−**0.75 (**−**1.06;** −**0.17)**	617	−0.60 (−1.50; 0.30)	631	−**0.39 (**−**0.77;** −**0.01)**
GÅS										
Basic model	1053	−0.07 (−0.23; 0.09)	1100	−0.14 (−0.30; 0.02)	1042	−**0.19 (**−**0.34;** −**0.03)**	783	**0.37 (0.15; 0.59)** ^*^	1037	−**0.17 (**−**0.29;** −**0.06)**
Fully adjusted model	1053	0.02 (−0.17; 0.20)	1100	−0.10 (0.28; 0.08)	1042	**−0.14 (**−**0.31;** −**0.03)**	783	**0.24 (0.01; 0.47)** ^*^	1037	−0.10 (−0.22; 0.03)
SNAC-K										
Basic model	1456	−**0.34 (**−**0.64;** −**0.04)**	1485	−**0.31 (**−**0.62;** −**0.01)**	1410	−0.23 (−0.53; 0.08)	1142	**0.76 (0.36; 1.16)** ^*^	1468	−**0.40 (**−**0.62;** −**0.18)**
Fully adjusted model	1456	−0.25 (−0.55; 0.05)	1485	-0.21 (-0.51; 0.08)	1410	−0.13 (−0.43; 0.16)	1142	**0.64 (0.24; 1.04)** ^*^	1468	−**0.30 (**−**0.51;** −**0.08)**

These estimates are produced from model 1. Basic model controlling for age, sex, education and model 2. Fully adjusted model controlling for age, sex, education hypnotics, smoking, living alone, alcohol consumption, physical inactivity, depressive symptoms, stroke, myocardial infarction, atrial fibrillation, diabetes, heart failure, and hypertension. If the models presented a significant result in a study for any of the sleep parameters, then the estimate is presented in this table, together with the other domain estimates for that study. No other nonsignificant results are presented here. For clarity of which significant association could have potentially been observed, the studies with corresponding sleep parameters are presented in parenthesis.

Bold typeface denotes *p* < 0.05.NA: Not applicable.

^*^These estimates are based on speed tests, where a positive estimate, i.e., taking longer time to complete the test, is equal to a poorer performance.

Abbreviations: SNAC-K: Swedish National Study on Aging and Care- Kungsholmen: SNAC-GÅS: Swedish National Study on Aging and Care – Good Aging in Skåne; H70: The Gothenburg H70 Birth Cohort Studies; CAIDE: The Cardiovascular Risk Factors, Aging and Dementia study; KP: The Kungsholmen Project.

**Table 4.** Mixed model coefficient estimates for longitudinal associations with significant results for sleep parameter and cognitive domain in each study. Comparison of estimates from two models, one basic model and one fully adjusted model

	Cognitive domains									
	Episodic memory		Verbal fluency		Perceptual speed (5)		Executive functioning (3)		Global cognition (2)	
Sleep parameter (studies with parameter)	*n*	Estimation (95% CI)	*n*	Estimation (95% CI)	*n*	Estimation (95% CI)	*n*	Estimation (95% CI)	*n*	Estimation (95% CI)
*Sleep latency (available in H70, GÅS, SNAC-K)*										
SNAC-K										
Basic model	1456	−0.01 (−0.04; 0.01)		−0.01 (−0.03; 0.01)		−0.01 (−0.03; −0.01)		**0.03 (0.00; 0.06)** ^1^		−0.01 (−0.03; 0.00)
Fully adjusted model	1456	−0.01 (−0.04; 0.01)		−0.01 (−0.03; 0.01)		−0.01 (−0.03; −0.01)		**0.03 (0.00; 0.06)** ^1^		−0.01 (−0.03; 0.00)
*Waking up at night (available in H70, GÅS, SNAC-K)*										
GÅS										
Basic model	1053	−**0.02 (**−**0.04;** −**0.00)**	1100	−0.01 (−0.03; 0.01)	1042	−0.02 (−0.04; 0.00)	783	−0.00 (−0.03; 0.03)^1^	1037	−0.01 (−0.03; 0.01)
Fully adjusted model	1053	−**0.02 (**−**0.04;** −**0.00)**	1100	−0.02 (−0.04; 0.00)	1042	−0.02 (−0.04; 0.00)	783	0.00 (−0.03; 0.03)^1^	1037	−0.01 (−0.02; 0.01)
Early awakenings (available in H70, GÅS, SNAC-K, CAIDE)										
GÅS										
Basic model	1053	−0.02 (−0.05; 0.01)	1100	−0.00 (−0.03; 0.02)	1042	−**0.04 (**−**0.06;** −**0.01)**	783	0.04 (−0.01; 0.08)^1^	1037	−0.02 (−0.05; 0.00)
Fully adjusted model	1053	−0.01 (−0.04; 0.02)	1100	−0.01 (−0.04; 0.02)	1042	−**0.03 (**−**0.06;** −**0.00)**	783	0.04 (−0.01; 0.08)^1^	1037	−0.02 (−0.04; 0.00)
*Sleep duration (available in H70 & GÅS)*										
Short sleep										
GÅS										
Basic model	1053	−0.01 (−0.03; 0.04)	1100	−0.02 (−0.05; 0.01)	1042	−**0.02 (**−**0.04;** −**0.01)**	783	**0.03 (0.01; 0.06)** ^*^	1037	−0.02 (−0.05; 0.00)
Fully adjusted model	1053	−0.00 (−0.02; 0.03)	1100	−0.00 (−0.02; 0.01)	1042	−**0.02 (**−**0.04;** −**0.01)**	783	**0.03 (0.00; 0.06)** ^*^	1037	−0.01 (−0.03; 0.00)
Long sleep										
GÅS										
Basic model	1053	−0.01 (−0.03; 0.04)	1100	−0.02 (−0.05; −0.01)	1042	−**0.04 (**−**0.07;** −**0.01)**	783	0.02 (−0.02; 0.10)^*^	1037	−0.02 (−0.04 0.00)
Fully adjusted model	1053	−0.01 (−0.03; 0.04)	1100	−0.02 (−0.05; −0.01)	1042	−**0.04 (**−**0.07;** −**0.01)**	783	0.02 (−0.02; 0.10)^*^	1037	−0.02 (−0.04 0.00)
*Daytime napping (available in H70, GÅS, SNAC-K)*										
SNAC-K										
Basic model	1456	−0.02 (−0.09; 0.05)	1485	−**0.06 (**−**0.12;** −**0.00)**	1410	−**0.07 (**−**0.13;** −**0.02)**	1142	**0.02 (**−0.11; 0.08)^*^	1468	−**0.05 (**−**0.10;** −**0.01)**
Fully adjusted model	1456	−0.02 (−0.09; 0.04)	1485	−**0.06 (**−**0.12;** −**0.00)**	1410	−**0.08 (**−**0.13;** −**0.02)**	1142	−0.01 (−0.11; 0.08)^*^	1468	−**0.06 (**−**0.10;** −**0.01)**
GÅS										
Basic model	1053	−**0.05 (**−**0.08;** −**0.01)**	1100	−**0.04 (**−**0.07;** −**0.01)**	1042	−**0.07 (**−**0.10;** −**0.04)**	783	0.04 (−0.01; 0.10)^*^	1037	−**0.05 (**−**0.08;** −**0.03)**
Fully adjusted model	1053	−**0.06 (**−**0.09;** −**0.02)**		−**0.03 (**−**0.07;** −**0.00)**		−**0.07 (**−**0.10;** −**0.03)**		**0.06 (0.01; 0.12)** ^*^		−**0.06 (**−**0.08;** −**0.03)**

These estimates are produced from model 1. Basic model controlling for age, sex, education and model 2. Fully adjusted model controlling for age, sex, education hypnotics, smoking, living alone, alcohol consumption, physical inactivity, depressive symptoms, stroke, myocardial infarction, arterial fibrillation, diabetes, heart failure, and hypertension. If the models presented a significant result in a study for any of the sleep parameters, then the estimate is presented in this table, together with the other domain estimates for that study. No other nonsignificant results are presented here. For clarity of which significant association could have potentially been observed, the studies with corresponding sleep parameters are presented in parenthesis.

Bold typeface denotes *p* < 0.05.

^*^These estimates are based on speed tests, where a positive estimate, i.e. taking longer time to complete the test, is equal to a poorer performance. NA: Not applicable.

Abbreviations: SNAC-K: Swedish National Study on Aging and Care- Kungsholmen; SNAC-GÅS: Swedish National Study on Aging and Care – Good Aging in Skåne; H70: The Gothenburg H70 Birth Cohort Studies; CAIDE: The Cardiovascular Risk Factors, Aging and Dementia study; KP: The Kungsholmen Project.

#### Reduced sleep duration/quality (available in H70, GÅS, SNAC-K, KP).

Participants reporting reduced sleep duration/quality performed significantly worse on tests measuring verbal fluency in KP, and this association was attenuated to nonsignificant when controlling for hypnotics. No significant longitudinal associations were detected.

#### Sleep latency—problems falling asleep (available in H70, GÅS, SNAC-K).

Cross-sectional associations between sleep latency and worse scores on tests measuring episodic memory and global cognition were observed in the fully adjusted models in the H70-study. Likewise, in H70, problems falling asleep were associated with verbal fluency, perceptual speed, and executive functioning; however, the associations were reduced to nonsignificant when controlling for hypnotics and depression. Longitudinally, participants reporting issues with falling asleep in the SNAC-K study performed worse over time on tests measuring perceptual speed, executive function, and global cognition.

#### Waking up at night (available in H70, GÅS, SNAC-K).

In the H70-study, among participants reporting waking up once or more during the night, lower level of performance was observed on episodic memory, verbal fluency tests, perceptual speed, and global cognition. The association for episodic memory and perceptual speed became nonsignificant when adjusting for hypnotics. Longitudinally, faster decline in episodic memory was observed for participants reporting multiple awakenings in the GÅS study.

#### Early awakenings (available in H70, GÅS, SNAC-K, CAIDE).

In the H70-study, participants reporting early awakenings demonstrated lower level of performance on tests measuring all cognitive domains, including global cognition. Although, excluding episodic memory, associations for verbal fluency, perceptual speed, executive functioning, and global cognition were attenuated to nonsignificant when adjusting for hypnotics. Furthermore, participants in the CAIDE study reporting early awakenings had lower global cognition scores but the significant association estimates no longer remained when controlling for lifestyle factors. Over time, perceptual speed performance declined faster for participants in the GÅS study reporting waking up early.

#### Sleep duration (available in GÅS and H70).

In the basic models adjusted for demographics, participants sleeping 6 hours or less in comparison to sleeping 7 to 8 hours per night showed lower level of performance on episodic memory tests in the H70 study. Associations were no longer significant when controlling for depression. The longitudinal analyses showed that participants sleeping 6 hours or less declined faster on tests measuring perceptual speed and executive function for GÅS participants.

For long sleep, in GÅS, sleeping for 9 hours or more, compared to participants sleeping for 7 to 8 hours, was associated with lower level of performance in all cognitive measures. All associations were attenuated to nonsignificant when controlling for cardiovascular conditions. In the H70-study, long sleepers performed worse on measures of perceptual speed. The longitudinal analyses showed poorer test scores over time for perceptual speed for long sleepers in comparison to those sleeping 7 to 8 hours in the GÅS study.

#### Excessive daytime napping (available in H70, GÅS, SNAC-K).

Cross-sectionally, feeling tired and taking naps for 2 hours or more during the day was associated with lower level of performance in episodic memory, verbal fluency, perceptual speed and global cognition in H70, and associated with poorer perceptual speed, executive functioning, and global cognition in GÅS, and poorer episodic memory, verbal fluency, executive functioning, and global cognition in SNAC-K. The associations with verbal fluency in H70, with global cognition in GÅS, and with episodic memory and verbal fluency in SNACK-K were reduced to nonsignificant in the fully adjusted model, when controlling for hypnotics, lifestyle factors, and depressive symptoms, respectively.

As for longitudinal associations, participants who reported daytime napping had steeper cognitive decline in all examined cognitive domains in GÅS, and in verbal fluency, perceptual speed, executive functioning, and global cognition in SNAC-K. Although, associations for executive functioning in the SNAC-K study were attenuated to nonsignificant when controlling for hypnotics. To provide an example of the magnitude of associations seen for test scores, an effect size of -0.06 for the longitudinal association for napping and episodic memory (in GÅS) is comparable to a faster decline in recall of 0.15 words per year (fully adjusted models).

## Discussion

This study aimed to explore cross-sectional and longitudinal associations between various sleep disturbances and cognitive domains in five Nordic cohorts of older adults using a coordinated analytic approach that maximized analysis of available data across studies. After controlling for demographic characteristics and other potential confounders of sleep and cognitive impairment, results showed that participants reporting insomnia symptoms, abnormal sleep duration, and excessive daytime napping performed worse on a range of cognitive tests and had steeper cognitive decline than those without subjective sleep disturbances. All the examined cognitive domains exhibited cross-sectionally and longitudinally associations with sleep disturbances in at least one cohort, with perceptual speed having the greatest number of significant associations. In general, results were study-specific; however, there were multiple equivalent associations observed for napping across three studies. This coordinated analysis produces more rigorous and comparable evidence to that of sequential results from independent studies, because of our implementation of an identical statistical model and covariate adjustments, while using a wide range of sleep and cognitive measures carefully selected to represent each parameter. This coordinated approach minimizes potential result heterogeneity due to both differences in measurement and statistical models.

The current results support previous work demonstrating that objective and subjective measures of insomnia are related to worse verbal fluency, episodic memory, executive functioning, and global cognition [18, 21, 22, 61]. Notably, the associated domain varied across studies, consistent with prior studies exhibiting conflicting associations for insomnia and cognitive domains [23–26, 62]. Methodological limitations, heterogeneous samples, varying, and unsuitable cognitive tests may be responsible for discrepancies among study results [62]. Yet, despite acquiring comparable, and in some instances identical, cognitive and sleep measurements were observed inconsistencies. For instance, in H70, participants with sleep latency issues and early awakenings demonstrated poorer episodic memory, which was not seen for waking up at night in the fully adjusted model. Whereas, GÅS participants expressing multiple night time awakenings had faster memory decline. Other factors for discrepancies may include differences in follow-up time, cohort characteristics not controlled for, the root causes of insomnia, as well as other factors potentially not controlled for. Moreover, although, our results suggest that symptoms of insomnia are only mildly associated with poorer cognitive performance in older adults, two recent studies showed a cross-sectional [27] and a longitudinal association [32] with insomnia disorder and memory, but no associations for insomnia symptoms only. Hence, experience of insomnia symptoms alone, especially when presented individually, may not be enough to impact cognition.

Our findings were consistent with prior studies supporting the u-shape theory of sleep duration and cognition [36–38, 63], where both short and long sleep are linked to cognitive impairment and decline. Cross-sectionally, longer sleep at baseline was associated with all cognitive domains, but only estimates for perceptual speed remained significant when controlling for health and lifestyle factors. Likewise, our longitudinal associations between sleep duration and the cognitive outcomes perceptual speed (for short and long sleep) and executive functioning (short sleep) are comparable to results from other studies [38, 64, 65]. A meta-analysis showed that extreme sleep durations were cross-sectionally related to measures of memory, but not to processing speed, and that faster decline was only seen for aggregated multiple domain measures [65]. Notably, the authors point out that the effect sizes for perceptual speed and sleep duration were comparable to the other significant associations and may be driven by the low number of studies on perceptual speed. Agreement in thresholds (cutoffs) for sleep durations is lacking, leading to inconsistencies in inferences. Periods of extreme sleep deprivation (<4 hours) or excessive sleep (>10 hours) may be more detrimental for cognition. For instance, a pooled analysis showed worse global cognition for those sleeping >10 hours, or for 5 hours per night in comparison to 7, but this was not seen for sleeping durations 6, 8, or 9 hours [36]. In the literature, excessive sleep is more strongly related to cognitive impairment, decline and dementia than deficient sleep [35, 63, 66, 67] and likewise, excessive daytime napping was steadily associated with lower cognitive functioning in our analyses.

In three of the viable studies, multiple cross-sectional and longitudinal associations were detected for excessive napping and cognition. Our findings are coherent with prior research reporting that extensive daytime napping and daytime sleepiness are associated with poorer episodic memory, executive functioning, perceptual speed, and measures of global cognition [21, 42, 43, 46, 68]. Most previous work on napping and cognitive decline examine global measures of cognition or incident dementia [42, 43, 69, 70] with few investigating individual cognitive domains. Two studies showed steeper decline in processing speed among nappers [68, 71], similar to our results. Our findings contribute with novel and consistent evidence showing longitudinal associations for excessive napping and decline in episodic memory, verbal fluency and executive functioning and strengthens existing cross-sectional evidence. Notably, in H70, only a few participants met the criteria for excessive daytime napping. This could explain why no longitudinal associations in H70 were observed and the observed significant findings in this study should be interpreted with caution. Daytime napping is both an indicator and symptom of cognitive impairment and dementia [42, 72]. We excluded those with MMSE scores <24 and dementia at baseline. Nevertheless, we cannot rule out that excessive napping could be an early dementia marker, as disease pathology may exist years prior to clinical symptoms.

Perceptual speed was the domain mostly related to sleep disturbances, followed by episodic memory and executive functioning. Some [38, 68, 73, 74], but not all [18, 23, 65, 75] studies report that objective and subjective measures of sleep disturbances are related to worse perceptual speed. Furthermore, characteristics and the purity of perceptual speed test (in SNAC studies) can provide some clarification, as they are easy to perform with little requirement of competing cognitive abilities making them extra susceptible to age and health-related change [76]. Furthermore, worse white matter integrity in various brain areas has been linked to both short sleep and worse speed of processing, serving as potential mediator for our observed associations [77, 78]. Nevertheless, whether speed is especially susceptible to poor sleep requires further replication especially as the results are mixed. Multiple studies show worsened episodic memory and executive functioning for a range of sleep disturbances [18, 37, 61, 65, 73]. Deficits in these domains together with perceptual speed are also observed in the preclinical stages of dementia [79, 80]. Although dementia cases were excluded at baseline, observed longitudinal associations could be in part due to early dementia-related changes.

Underlying medical, pharmacological and psychiatric conditions, or neurodegenerative processes may explain how sleep and cognition in older adults are linked. For instance, tau-pathology in wake-promoting neurons cause sleep-wake disturbances and napping tendencies [81] or insufficient beta-amyloid protein clearance due to sleep deficiency [82, 83]. Both tau-pathology and amyloid accumulation are key features of AD. Moreover, sleep disturbance is suggested to elicit neuroinflammation leading to disrupted neurogenesis, and when this occurs in hippocampal areas, learning and memory are likely to be impaired [84–86]. A recent study also showed that normal sleep duration (6 to 8 hours) was associated with greater gray matter volume in 33% of the investigated brain regions, including the frontal, temporal, parietal, and cerebellar regions [64].

Daytime napping can be a feature of insufficient night time sleep and may share the same underlying causes as sleep deprivation. In addition, primary health issues could act as modifiers explaining cognitive impairment in long sleepers. Evidence shows that the presence of comorbidities (e.g. cardiovascular disease, diabetes, and depression) increases the likelihood of excessive sleep (daytime napping and long night time sleep) [2, 87] and cognitive impairment [88]. Yet, only 26.3% of our observed napping results were explained by covariates such as depression and cardiovascular disease. Although, all cross-sectional associations for long-sleep and for all cognitive domains were no longer significant when adjusting for cardiovascular and metabolic factors in the GÅS study. Nevertheless, similar discrepancies regarding the role of health-related modifiers are found across observational studies and notably far from all studies control for suitable cofounders. Furthermore, sleep disordered breathing is fairly prevalent among older adults and is linked to sleep disruption, cognitive impairment, and dementia [3, 89] and could explain some of our results.

Disparities in the observed sleep-cognition associations across our studies are equivalent to what is seen in the literature [10]. Nevertheless, none of the significant estimates pointed in the direction that those reporting sleep disturbances performed better on cognitive tests. Moreover, despite common methodological concerns in aging studies, such as increased variability in cognitive test scores and sleep disturbances, or retest effects, we still observe a consistent pattern of associations. In addition, research efforts from European Nordic countries, with some of the current included studies, similarly show that sleep disturbances and midlife nightmares were associated with lower MMSE scores, and dementia risk was increased for those reporting midlife insomnia and late-life early awakenings or long sleep duration [6, 29].

A major strength of this study is that it examined a range of sleep parameters and cognitive domains, that were not all available in each study, using large datasets from several cohorts of older adults in Northern Europe, enhancing the generalizability of the results. This set of analyses also provides new results on longitudinal changes in cognition, based on at least three follow-ups in four of the five studies examined. We also adjusted for several potential confounders. There are a few limitations worth mentioning. First, given our study design, no causal conclusions can be determined, and there is also a possibility of reversed causality, where early cognitive impairment could cause sleep disturbances. For instance, one study observed a bidirectional relationship between napping and cognition [42]. Other experimental studies, point to a causal association for dementia, where disrupted sleep increased AD risk through increased production of beta-amyloid [83, 90]. Second, despite our efforts to select identical sleep questions, they differed slightly in both wording and execution (self-reported or blocked questions), which may partly explain the varying prevalence of sleep disturbances across studies (e.g. 40.9% reported waking up at night in the H70 study but only 18.0% in SNAC-K) and some of the observed heterogeneity in the results. Albeit, the prevalence of sleep disturbances in older adults vary substantially in the literature [91]. Moreover, our questions on napping does not differentiate if the participant experiences fatigue rather than sleepiness. More specific questions would clarify the specific roles of tiredness, sleepiness, fatigue, need for napping, and the different combinations of symptoms. Third, although inaccurate inferences are a potential problem due to multiple testing, all significant estimates were in the same direction, reducing the probability of merely representing chance findings. Fourth, we used self-reported sleep measures, and apart from differential misclassification risks, subjective versus objective sleep measures tend to produce conflicting results when tested against the same cognitive outcome [9, 92]. Hence, further studies including objective and subjective sleep parameters are needed. A further limitation includes potential birth-cohort effects contributing to the presence or absence of significant results between studies, i.e. the study cohorts were born on different years which can both affect cognition and sleep patterns in older adults [56, 93, 94]. However, as each study performed their own analysis, we were limited in controlling for these potential effects between studies. Finally, selection and attrition bias, where the cognitively impaired do not participate or drop out during the course of the study may underestimate the observed associations through lower effect sizes.

In summary, consistent with existing research, findings from this multicenter coordinated analysis provide evidence in favor that sleep and cognition is related in older adults. More specifically, the study-specific results showed that symptoms of insomnia are related to worse episodic memory. Also, short and longer night time sleep were associated with worse scores and steeper decline on measures of perceptual speed. Short sleep was additionally associated with poorer scores over time on executive functioning. Reciprocated results in three studies included that daytime napping was cross-sectionally and longitudinally related to almost all investigated cognitive domains. This pinpoints that specific sleep behaviors together with certain cognitive abilities could prove beneficial for interventions targeting specific negative outcomes (e.g. AD, which is characterized by memory deficits).

## Data Availability

For data within NEAR, data are available through the National E-infrastructure for Aging Research (NEAR, www.near-aging.se) for approved applicants. For CAIDE data, applications must be submitted to the CAIDE Steering Committee.
